# Enhancing the Temporal Complexity of Distributed Brain Networks with Patterned Cerebellar Stimulation

**DOI:** 10.1038/srep23599

**Published:** 2016-03-24

**Authors:** Faranak Farzan, Alvaro Pascual-Leone, Jeremy D. Schmahmann, Mark Halko

**Affiliations:** 1 Temerty Centre for Therapeutic Brain Intervention, Centre for Addiction and Mental Health, University of Toronto, ON, M6J 1H4, Canada; 2 Berenson-Allen Center for Noninvasive Brain Stimulation and Division of Cognitive Neurology, Department of Neurology, Beth Israel Deaconess Medical Center, Harvard Medical School, MA 02215, USA; 3 Ataxia Unit, Cognitive Behavioral Neurology Unit, Laboratory for Neuroanatomy and Cerebellar Neurobiology, Department of Neurology, Massachusetts General Hospital, Harvard Medical School, MA 02114, USA

## Abstract

Growing evidence suggests that sensory, motor, cognitive and affective processes map onto specific, distributed neural networks. Cerebellar subregions are part of these networks, but how the cerebellum is involved in this wide range of brain functions remains poorly understood. It is postulated that the cerebellum contributes a basic role in brain functions, helping to shape the complexity of brain temporal dynamics. We therefore hypothesized that stimulating cerebellar nodes integrated in different networks should have the same impact on the temporal complexity of cortical signals. In healthy humans, we applied intermittent theta burst stimulation (iTBS) to the vermis lobule VII or right lateral cerebellar Crus I/II, subregions that prominently couple to the dorsal-attention/fronto-parietal and default-mode networks, respectively. Cerebellar iTBS increased the complexity of brain signals across multiple time scales in a network-specific manner identified through electroencephalography (EEG). We also demonstrated a region-specific shift in power of cortical oscillations towards higher frequencies consistent with the natural frequencies of targeted cortical areas. Our findings provide a novel mechanism and evidence by which the cerebellum contributes to multiple brain functions: specific cerebellar subregions control the temporal dynamics of the networks they are engaged in.

Organized neural activity serves as the biological foundation for human brain function. For efficient information processing, the brain temporal dynamics should not be completely predictable, nor should they be completely random^1^. The complexity of brain signals has been closely linked with cognitive capacity, efficiency of information processing, and behavioral stability^2^,^3^,^4^,^5^. Furthermore, loss of complexity has been associated with cognitive decline in aging and pathological conditions^2^,^3^.

Despite its small size, the cerebellum contains more than seventy percent of all neurons in the brain and is critically involved in a host of brain functions and impairments implicating sensory, motor, cognitive and affective processes^6^,^7^,^8^,^9^,^10^,^11^,^12^. The repeating modular cortical architecture of the cerebellum suggests that it may serve a domain-general role across brain functions^9^,^13^. It is suggested that the cerebellum may serve a fundamental role by modulating temporal dynamics essential for timing in information processing^14^,^15^,^16^,^17^,^18^. Indeed, simple and complex spikes of Purkinje cells of the cerebellar cortex control the temporal patterns generated by the inferior olive^17^,^18^.

On the other hand, neuroimaging and clinical investigations involving lesions to cerebellar subregions reveal a domain-specific cerebellar organization, with anterior cerebral regions mainly involved in motor, and posterior regions in cognitive control^7^,^9^. Anterograde, retrograde and transneuronal tract tracing techniques in primates revealed that prefrontal and other association cortices are reciprocally interconnected with different subregions of cerebellum^19^,^20^,^21^. In humans, task-based fMRI studies have provided evidence for task-specific activation of cerebellar subregions^7^,^8^. Consistent with task-specific activation^22^, resting-state functional connectivity MRI revealed an intrinsic functional connectivity between cerebellar subregions and major brain networks with hubs in the cerebrum^23^. For example, within the posterior cerebellar regions, the right lateral cerebellum is active during language processing^24^ and the right lateral Crus I/II functionally coupled with the default-mode network. In contrast, cerebellar vermis is implicated in selective attention and cognitive affective processing and functionally coupled with the dorsal-attention and fronto-parietal control networks^7^,^23^.

Therefore, cerebellar subregions may be critical network nodes governing complex temporal patterns in the distributed neural networks that each is engaged in. Here, we examined whether cerebellar stimulation of different network nodes within cerebellum leads to a general increase in the complexity of the brain signals, and whether this increase is prominently observed in cortical areas corresponding to the network stimulated through cerebellum. We used EEG to record cortical temporal dynamics and iTBS to upregulate activity of two posterior cerebellar regions^25^,^26^. We employed multiscale entropy (MSE) to assess the complexity and variability of brain signals across multiple time scales. The MSE metric quantifies the complexity of information carried by a biological signal across multiple time scales such that a purely deterministic or random signal would have a low complexity index and a structurally rich signal a high index^4^. We also examined the influence of cerebellar iTBS on the spectral characteristics of cortical activities. We predicted that if cerebellum is involved in generation of complex temporal patterns, stimulation of both cerebellar regions would lead to an increase in MSE at all time scales.

## Material and Methods

### Subjects

We studied ten healthy subjects (mean age: 30 ± 10 yr, 5 females). All had normal neurological and general medical examinations, and met criteria for minimizing the risk of TMS^27^. Exclusion criteria included a self-reported medical illness and history of seizure, neuropsychiatric disorders, drug or alcohol abuse, and any ongoing medications. All participants gave their written informed consent and the protocol was approved by the local ethics committee at the Beth Israel Deaconess Medical Center in accordance with the declaration of Helsinki.

### Design

**Figure 1:**
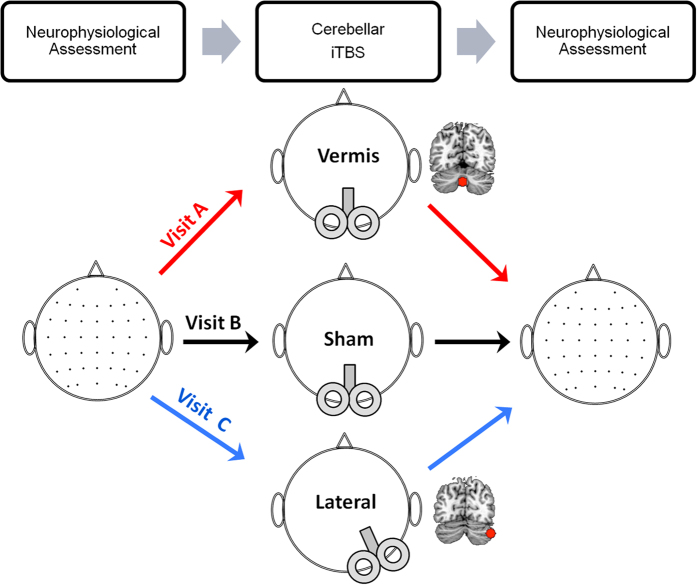
Study Design. EEG neurophysiological assessments were conducted before and after three visits involving application of active intermittent theta burst stimulation (iTBS) to cerebellar vermis (lobules VIIAt/VIIB), lateral cerebellum (the default-mode network node in Crus I/II), and sham to the vermis.

The study involved four visits: First, participants underwent a structural and resting-state functional MRI to neuronavigate iTBS placement. Visits 2–4 were separated by at least 48 hours and involved EEG neurophysiological assessments before and after cerebellar iTBS (Fig. 1).

### Cerebellar iTBS

#### Protocol

The iTBS was delivered through MagPro X100 using a double-blind Active/Placebo (AP) Cool-B65 figure-of-eight coil (MagVenture) coupled with neuronavigation (Brainsight Rouge Research). The iTBS^28^ involves three biphasic waveform pulses at 50 Hz applied at 5 Hz, every 10 s for 600 pulses. At the beginning of visits, participant’s resting and active motor threshold (MT) were measured for the first dorsal interosseous (FDI) muscle in the motor cortex through electromyography (EMG) recordings (PowerLab AD Instruments) according to TMS standards^29^. The intensity of cerebellar iTBS was 100% of active MT (AMT) according to our previous studies^26^,^30^. AMT was achieved by instructing subjects to maintain a contraction roughly equivalent to 20% of their maximum voluntary contraction force. Then AMT was defined as the intensity that resulted in an MEP of amplitude greater than 200 μv in 5 out of 10 trials as monitored with surface EMG electrodes using PowerLAB (PowerLab AD Instruments). Muscle contraction was monitored online with surface EMG and participants were reminded to maintain the same force if a change in magnitude of background noise was observed.

#### Localization

Lateral iTBS was applied to right lateral cerebellar Crus I/II, a region linked with the default-mode network^23^,^26^,^31^. Vermis iTBS targeted lobules VIIA/VIIB (mean MNI: 1, −73, −33)^30^,^32^, the most posterior and medial region of cerebellum, linked with dorsal-attention or fronto-parietal network^7^,^23^,^26^,^31^. The sham condition was applied to vermis. During all conditions (active or sham), two shamming surface electrodes were placed near the stimulation site. The shamming electrodes were Ambu neuroline 710, placed on the skin within 2 mm of each other on opposite sides of midline below the hairline. They are placed close enough together to shunt across the skin to simulate the experience of TMS. The coil was equipped with a voltage potentiometer set at 50%, corresponding to 2–4 mA. Electrical current stimulation produced twitches in synchronization with the stimulation to mimic similar tactile sensation as active stimulation. In all conditions, the coil was oriented vertically, along the superior-posterior plane of the neck, with the handle facing upward. Subjects were blind to the conditions.

### EEG

Three minutes of EEG was sampled immediately before and after iTBS. Subjects were instructed to sit in an armchair with eyes closed. EEG recording was through a 32-channel EEG system (BrainProducts, GmbH) with the CPZ and AFZ electrodes set as reference and ground electrode, respectively. EOG was recorded through two channels placed underneath each eye. The sampling rate was 5 kHz. The online filter setting was DC to 1 kHz. The skin/electrode impedance was kept below 5 kOhm.

### Data Analysis

#### EEG Preprocessing

Data were imported into MATLAB (The MathWorks. Inc. Natick). The EEGLAB toolbox version 11b^33^ was used for import and preprocessing. The signals were down sampled to 2 kHz and epoched into 2 seconds segments. Epochs and channels containing non-physiological artifact were discarded. A notch filter (band-stop: 55–65 Hz) was used to remove the 60 Hz noise. Signals were band-pass filtered for 1–50 Hz. The second order infinite impulse response Butterworth forward and backward filtering was applied (MATLAB function ‘filtfilt’). Independent component analysis was employed to remove eye movements, blinks, and EMG artifact. Missing channels were interpolated and the data were average re-referenced.

#### Multi-Scale Entropy

**Figure 2:**
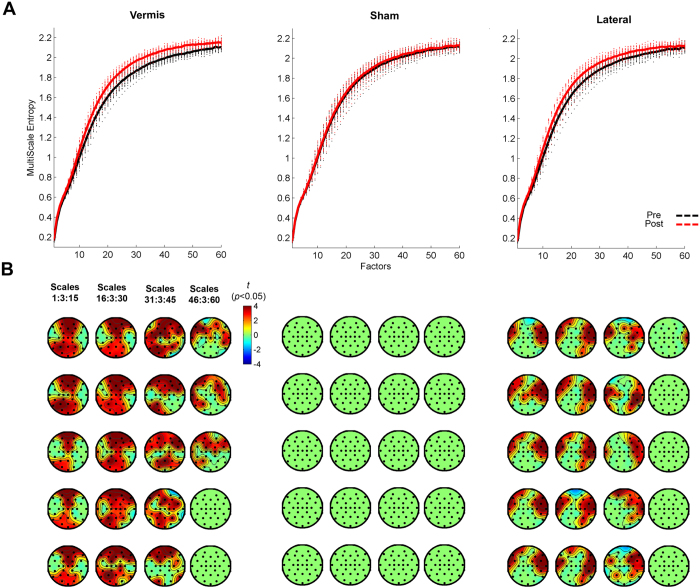
Effect of Cerebellar iTBS on Complexity of Temporal Dynamics. (**A**) MSE before (black line) and after (red line) vermis, sham and lateral cerebellar iTBS. The lines represent the average MSE across electrodes (dots). (**B**) Paired t-test maps comparing MSE post iTBS to pre across electrodes and scales 1–60, illustrated for every other 3 scales. All corrected non-significant t values are set to zero.

We examined MSE across electrodes (Fig. 2A). The MSE calculation included two steps^4^: The *coarse-graining* process and the calculation of the *SampEn*. In the first step, for a given time series 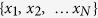, the multiple coarse-grained time series 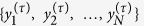
at scale factor τ are calculated by averaging the data points within non-overlapping windows of increasing length τ. Each element of the coarse-grained time series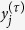 is calculated according to the equation 1:


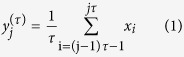


where τ represents the scale factor and 
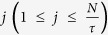
 represents the time index. The length of each coarse-grained time series is M, where M = floor 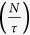. At scale factor τ = 1, the coarse-grained time series is the original time series. In the second step, the degree of predictability is measured for each of the multiple coarse-grained time series 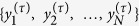 using SampleEn calculated as equation 2:


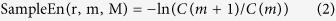


where C(m) is the total number of pairs of *m* consecutive similar data points, and C(m + 1) is the total number of pairs of m + 1 consecutive similar data points in the multiple coarse-grained time series. SampleEn quantifies the variability of time series by estimating the predictability of amplitude patterns across a time series. We used two consecutive data points (m = 2) for data matching and data points were considered to match if their absolute amplitude difference was less than 0.15% (r = 0.15) of standard deviation of time series. We applied MSE to two 12 s (6 consecutive epochs) time segments.

#### Power

**Figure 3:**
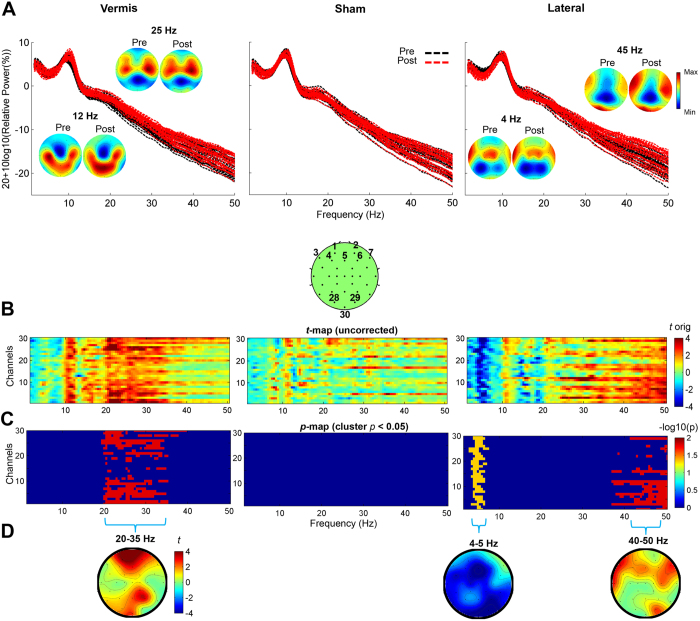
Effect of Cerebellar iTBS on Frequency of Cortical Oscillations. (**A**) Relative power spectrum across electrodes pre (black lines) and post (red lines) iTBS in vermis, sham and lateral conditions. Topographic plots illustrate the spatial pattern of the power spectrum for selected frequencies. (**B**) Parametric paired t-test maps comparing the relative power across frequency bands (x-axes) and channels (y-axes) post iTBS to pre in vermis, sham and lateral conditions (blue: decreases; red: increases following TMS). (**C**) Each image reflects the statistical significance (−log10 of *p* values) of the t-maps depicting only the significant clusters with *p* < 0.05, setting to 0 non-significant pixels. (**D**) Topographies highlight the spatial characteristics of the increase in power of beta to low gamma oscillations in frontal and parietal regions following vermis stimulation, and a global reduction in theta and an increase in high gamma oscillations in fronto-temporal areas following lateral stimulation. No significant changes are observed following sham.

We obtain the power spectrum across electrodes (Fig. 3A). Relative power was calculated as the ratio in the power of each frequency relative to the sum of power across all frequencies (1–50 Hz).

### Statistics

A cluster-based non-parametric permutation test^34^ was used to examine the effect of iTBS on MSE and relative power for main effects of Conditions (Vermis, Sham and Lateral stimulation) and Time (Pre, Post). This was used to correct for the multiple comparisons in this multi-dimensional dataset (30 channels × 50 frequencies, 30 channels × 70 scales) by assigning significance statistics to the size of clusters formed by pooling neighboring pixels with original test statistics *p* < 0.05. Analysis of variance and post-hoc paired t-test analyses were used to calculate the original test statistics. The significance of clusters (cluster *p* < 0.05) was defined using 1000 permutations.

## Results

### The effect of cerebellar iTBS on the complexity of temporal dynamics

As predicted, we found a main effect of Condition on MSE (corrected *p* < 0.05). Post-hoc analysis revealed that both the vermis and lateral iTBS increased MSE (corrected *p* < 0.05). Sham stimulation did not change the MSE. No other comparisons were significant (Fig. 2B).

### The effect of cerebellar iTBS on the frequency of cortical oscillations

We found a main effect of Time, Condition, and an interaction effect of Time X Condition (corrected *p* < 0.05). Paired-wise analysis revealed a significant change in relative power towards higher frequencies following vermis and lateral stimulation compared to baseline (corrected *p* < 0.05). No changes were observed in sham (Fig. 3B).

### Network-specificity of the cerebellar iTBS effects

To assess if the iTBS-related modulation of signal complexity and power are specific to the functional networks that each cerebellar node engage in we examined their topographical distribution.

**Figure 4:**
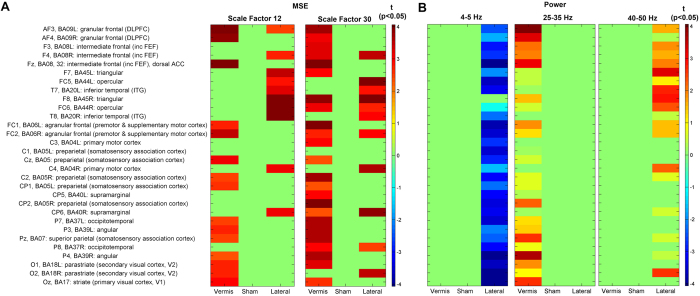
Network-Specific Effects of Cerebellar iTBS. (**A**) T-test maps depicting significant changes in MSE for two example scales (12 and 30) in vermis, sham and lateral conditions. Vermis and lateral conditions increased MSE in overlapping but different cortical regions. (**B**) T-test maps depicting significant changes in spectral power (blue: decreases; red: increases following TMS). Lateral iTBS reduced theta oscillations globally. Both vermis and lateral iTBS enhanced higher frequencies. Vermis increased power of 25–35 Hz mainly in frontal leads (DLPFC, FEF, and parietal regions), while lateral iTBS increased gamma oscillations (40–50 Hz) mainly in fronto-temporal areas. In both panels only t-values are shown that survived correction for multiple comparisons, and non-significant areas are set to 0 (green).

The topography of complexity modulation was different between conditions (Figs 2B and 4A). Vermis iTBS increased complexity in fronto-parietal leads corresponding to bilateral dorsolateral prefrontal cortex (DLPFC), dorsal anterior cingulate cortex (dACC), frontal eye field (FEF), and parietal regions. In contrast, lateral iTBS increased complexity in bilateral fronto-temporal regions (Fig. 2B), in leads corresponding to bilateral inferior and middle temporal gyrus and triangular and opercular gyri.

The vermis iTBS increased the power of high beta to low gamma oscillations (20–35 Hz) (Fig. 3B) mainly identified in the fronto-parietal regions including leads corresponding to bilateral DLPFC(Fig. 4B). The lateral iTBS significantly decreased relative power of theta (4–5 Hz) oscillations globally and increased gamma (40–50 Hz) oscillations (Fig. 3B) in the fronto-temporal leads (Fig. 4B).

## Discussion

We integrated and examined the notions of a *domain-general* role of cerebellum in shaping the brain temporal dynamics with the empirical evidence in support of *network-specific* cerebellar topography. The complexity of biological time series is postulated to reflect the plasticity of the system to an ever-changing environment and its adaptability to stressors^3^,^35^. A more variable and complex brain signal may produce more stable behavior^2^. A widespread increase in brain signal complexity has been observed during maturation^36^ whereas a spatiotemporally specific decline in complexity was observed in aging^3^. Moreover, complexity was increased during cognitive performance, importantly during cognitive control tasks^5^. Therefore, the network-specific but general increase in complexity following one session of cerebellar iTBS suggests that network-guided cerebellar iTBS can provide a novel approach for modulating a wide range of specific brain functions.

Indeed, morphometric and functional brain imaging studies identify a cerebellar contribution to such disparate conditions as attention deficit disorder^37^,^38^, depression^39^,^40^, linguistic processing^41^,^42^ and social emotional functioning^43^,^44^, in addition to its better known role in the ataxias^45^. Indeed, cerebellar stimulation with TMS appears to show promising results in some of these disorders^30^,^46^,^47^,^48^,^49^. Therefore our findings provide a new empirical explanation as to why and how targeting functionally relevant subregions in the cerebellum has the potential to have significant and clinically meaningful outcomes.

Interestingly, recent investigations have also revealed impairments of brain temporal complexity in disorders of cognition and affect such as autism spectrum disorder and Alzheimer’s disease (e.g.,^50^,^51^). The temporal complexity of brain signals is suggested to have a fundamental role in shaping the system’s capacity in processing information^4^,^52^,^53^,^54^. This complexity is linked to transient increases and decreases in correlated activity among local and distributed brain regions, subserving integration and segregation of information locally and globally^3^,^54^, reflecting plasticity of the brain in an ever changing environment^3^,^35^. A recent fMRI study using data from Human Connectome Project assessed the relationship between MSE applied to fMRI signals and resting-state functional connectivity across several resting-state networks and showed differential association between functional connectivity and complexity in fine versus coarse time scales^55^. Even though the time scales used in this fMRI study are much coarser compared to our EEG study making a direct comparison impossible, this study nevertheless provides evidence in support of an indirect but possible link between MSE and functional connectivity that warrants further investigation in future studies.

The finding that vermal stimulation increased the power of high beta/low gamma oscillations in fronto-parietal regions is in line with connectivity of this cerebellar region with dorsal-attention and fronto-parietal control networks^23^. Distinct brain landscapes oscillate at a peak natural frequency, likely due to differential neuronal composition and connectivity with other brain areas^56^. This is evident in Fig. 3A which illustrates relative predominance of theta oscillations in fronto-temporal, alpha in occipital, beta in parietal, and gamma in fronto-temporal areas. The reduction in theta oscillations following lateral stimulation is consistent with previous findings that activation of default-mode network leads to a reduction in frontal theta activity^57^. Moreover, due to the intrinsic amplitude modulation of gamma oscillations by theta, a reduction in theta oscillations is expected to lead to an increase in relative gamma. The increase in gamma oscillations in lateral frontal and temporal regions can also be linked with the suggested association of right lateral cerebellar regions in language processing which is associated with increased gamma in inferior frontal and temporal gyrus^58^.

We previously showed that cerebellar iTBS enhanced cortical resting-state functional connectivity: the default-mode network following lateral cerebellar stimulation, and the dorsal-attention network with vermal stimulation^26^. The present results provide compelling evidence in support of a network-specific but otherwise common temporal effect of posterior cerebellar activation. These observations are fully consistent with the dysmetria of thought theory^9^,^13^ in which the repeating cortical architecture of the cerebellum facilitates a domain general computation applied to multiple functionally distinct networks subserved through topographically precise cerebral, brainstem and spinal cord connections.

Our study complements previous studies that examined the effect of cerebellar iTBS on local excitation and inhibition of motor cortex or the long-range inhibitory effect of cerebellum on the motor cortex (e.g., reviewed in^59^). As examples, a previous study applied iTBS to posterior and superior lobules of lateral cerebellum and examined the change in local excitation and inhibition of the motor cortex^25^. It was shown that iTBS, applied at 80% of AMT to lateral cerebellum, increased the amplitude of MEPs and reduced long interval cortical inhibition in the motor cortex. In another study^60^ iTBS applied at 80% of AMT was shown to not change the magnitude of cerebellar inhibition, while cTBS reduced the long range cerebellar inhibition of motor cortex. It is suggested that a suprathreshold single pulse TMS applied to cerebellum activates Purkinje cells in the cerebellar cortex and subsequently inhibits the facilitatory cerebello(dentate nucleus)-thalamic efferent to the contralateral motor cortex. It is however postulated that the subthreshold intensity of cerebellar TBS may exert a differential effect. Subthreshold iTBS may engage low threshold local interneurons and indirectly activate dentate nucleus and have a facilitatory effect on the motor and association cortices^59^. The results of our study suggest that, irrespective of site of cerebellar stimulation, iTBS likely exerts similar plasticity changes in the cerebellum and therefore the corresponding brain network. Future studies should examine the specificity of findings to iTBS and whether cTBS has an opposite effect on complexity.

In summary, our results provide new evidence that cerebellar iTBS can enhance the complexity of temporal dynamics. We show that this effect follows a brain network structure, such that cerebellar iTBS to specific cerebellar subregions has the same fundamental impact on the cortical temporal dynamics but preferentially in cortical regions that functionally couple to these cerebellar subregions. These findings provide new mechanistic insight into how the cerebellum may be involved in a wide range of brain functions. This study provides early evidence to peruse several new questions such as the effect of repeated and daily cerebellar-induced enhancement of complexity on behavior, and the potential of cerebellar iTBS to enhance information processing in the treatment of brain disorders.

## Additional Information

**How to cite this article**: Farzan, F. *et al*. Enhancing the Temporal Complexity of Distributed Brain Networks with Patterned Cerebellar Stimulation. *Sci. Rep.* **6**, 23599; doi: 10.1038/srep23599 (2016).
